# Supplementary material for: Transcriptome Analysis Reveals Vimentin-Induced Disruption of Cell–Cell Associations Augments Breast Cancer Cell Migration
Source: Cells. 2022 Dec 13;11(24):4035. doi: 10.3390/cells11244035 (PMC9776984; doi:10.3390/cells11244035)
Supplement: Supplementary file 1 [file cells-11-04035-s001.zip › DEGs and enrichment analysis.zip!/DEGs and enrichment analysis/WT_VIMvsVector KEGG/GSEA/WT_VIM/NEUROACTIVE_LIGAND_RECEPTOR_INTERACTION/NEUROACTIVE_LIGAND_RECEPTOR_INTERACTION(HSA04080).html]

Details for gene set NEUROACTIVE\_LIGAND\_RECEPTOR\_INTERACTION(HSA04080)[GSEA]

|  || Dataset | fpkm.sample |
| Phenotype | sample.cls |
| Upregulated in class | WT\_VIM |
| GeneSet | NEUROACTIVE\_LIGAND\_RECEPTOR\_INTERACTION(HSA04080) |
| Enrichment Score (ES) | 0.6562144 |
| Normalized Enrichment Score (NES) | 1.1524526 |
| Nominal p-value | 0.0 |
| FDR q-value | 0.29252902 |
| FWER p-Value | 0.498 |
Table: GSEA Results Summary

  

Fig 1: Enrichment plot: NEUROACTIVE\_LIGAND\_RECEPTOR\_INTERACTION(HSA04080)      
 Profile of the Running ES Score & Positions of GeneSet Members on the Rank Ordered List

  

| PROBE | DESCRIPTION (from dataset) | GENE SYMBOL | GENE\_TITLE | RANK IN GENE LIST | RANK METRIC SCORE | RUNNING ES | CORE ENRICHMENT || 1 | ENSG00000011677 | GABRA3 |  |  | 48 | Infinity | -0.0008 | Yes |
| 2 | ENSG00000099957 | P2RX6 |  |  | 271 | Infinity | -0.0045 | Yes |
| 3 | ENSG00000139874 | SSTR1 |  |  | 302 | Infinity | -0.0050 | Yes |
| 4 | ENSG00000134489 | HRH4 |  |  | 323 | Infinity | -0.0053 | Yes |
| 5 | ENSG00000135577 | NMBR |  |  | 324 | Infinity | -0.0053 | Yes |
| 6 | ENSG00000064989 | CALCRL |  |  | 360 | Infinity | -0.0059 | Yes |
| 7 | ENSG00000180772 | AGTR2 |  |  | 361 | Infinity | -0.0058 | Yes |
| 8 | ENSG00000181408 | UTS2R |  |  | 426 | Infinity | -0.0069 | Yes |
| 9 | ENSG00000102195 | GPR50 |  |  | 480 | Infinity | -0.0078 | Yes |
| 10 | ENSG00000241563 | CORT |  |  | 505 | Infinity | -0.0081 | Yes |
| 11 | ENSG00000278195 | SSTR3 |  |  | 532 | Infinity | -0.0086 | Yes |
| 12 | ENSG00000121351 | IAPP |  |  | 599 | Infinity | -0.0096 | Yes |
| 13 | ENSG00000171596 | NMUR1 |  |  | 774 | Infinity | -0.0126 | Yes |
| 14 | ENSG00000257002 | AP000438.1 |  |  | 963 | Infinity | -0.0158 | Yes |
| 15 | ENSG00000186912 | P2RY4 |  |  | 1026 | Infinity | -0.0168 | Yes |
| 16 | ENSG00000233494 | AC009963.2 |  |  | 1148 | Infinity | -0.0188 | Yes |
| 17 | ENSG00000282608 | ADORA3 |  |  | 1192 | Infinity | -0.0195 | Yes |
| 18 | ENSG00000069696 | DRD4 |  |  | 1285 | Infinity | -0.0210 | Yes |
| 19 | ENSG00000160013 | PTGIR |  |  | 1403 | Infinity | -0.0230 | Yes |
| 20 | ENSG00000255082 | GRM5-AS1 |  |  | 1588 | Infinity | -0.0261 | Yes |
| 21 | ENSG00000178394 | HTR1A |  |  | 1676 | Infinity | -0.0276 | Yes |
| 22 | ENSG00000171873 | ADRA1D |  |  | 1678 | Infinity | -0.0275 | Yes |
| 23 | ENSG00000174697 | LEP |  |  | 1683 | Infinity | -0.0276 | Yes |
| 24 | ENSG00000160801 | PTH1R |  |  | 1878 | Infinity | -0.0309 | Yes |
| 25 | ENSG00000142538 | PTH2 |  |  | 1883 | Infinity | -0.0309 | Yes |
| 26 | ENSG00000138039 | LHCGR |  |  | 1947 | Infinity | -0.0319 | Yes |
| 27 | ENSG00000134817 | APLNR |  |  | 1992 | Infinity | -0.0327 | Yes |
| 28 | ENSG00000185231 | MC2R |  |  | 1998 | Infinity | -0.0327 | Yes |
| 29 | ENSG00000170893 | TRH |  |  | 2121 | Infinity | -0.0348 | Yes |
| 30 | ENSG00000182162 | P2RY8 |  |  | 2143 | Infinity | -0.0351 | Yes |
| 31 | ENSG00000166573 | GALR1 |  |  | 2187 | Infinity | -0.0358 | Yes |
| 32 | ENSG00000152578 | GRIA4 |  |  | 2188 | Infinity | -0.0358 | Yes |
| 33 | ENSG00000148734 | NPFFR1 |  |  | 2260 | Infinity | -0.0369 | Yes |
| 34 | ENSG00000239684 | PTGER4P3 |  |  | 2315 | Infinity | -0.0378 | Yes |
| 35 | ENSG00000180739 | S1PR5 |  |  | 2435 | 17.048 | 0.0117 | Yes |
| 36 | ENSG00000187730 | GABRD |  |  | 2557 | 8.920 | 0.0367 | Yes |
| 37 | ENSG00000109255 | NMU |  |  | 2588 | 8.407 | 0.0616 | Yes |
| 38 | ENSG00000164128 | NPY1R |  |  | 2622 | 7.770 | 0.0846 | Yes |
| 39 | ENSG00000126010 | GRPR |  |  | 2646 | 7.405 | 0.1066 | Yes |
| 40 | ENSG00000107018 | RLN1 |  |  | 2670 | 7.104 | 0.1277 | Yes |
| 41 | ENSG00000168539 | CHRM1 |  |  | 2847 | 5.731 | 0.1420 | Yes |
| 42 | ENSG00000078589 | P2RY10 |  |  | 2911 | 5.301 | 0.1570 | Yes |
| 43 | ENSG00000083454 | P2RX5 |  |  | 2932 | 5.247 | 0.1725 | Yes |
| 44 | ENSG00000135902 | CHRND |  |  | 3037 | 4.720 | 0.1851 | Yes |
| 45 | ENSG00000174944 | P2RY14 |  |  | 3053 | 4.645 | 0.1989 | Yes |
| 46 | ENSG00000127533 | F2RL3 |  |  | 3096 | 4.618 | 0.2121 | Yes |
| 47 | ENSG00000107014 | RLN2 |  |  | 3110 | 4.481 | 0.2255 | Yes |
| 48 | ENSG00000006638 | TBXA2R |  |  | 3128 | 4.438 | 0.2386 | Yes |
| 49 | ENSG00000125384 | PTGER2 |  |  | 3137 | 4.406 | 0.2518 | Yes |
| 50 | ENSG00000180210 | F2 |  |  | 3166 | 4.318 | 0.2644 | Yes |
| 51 | ENSG00000123901 | GPR83 |  |  | 3197 | 4.298 | 0.2769 | Yes |
| 52 | ENSG00000069482 | GAL |  |  | 3209 | 4.229 | 0.2895 | Yes |
| 53 | ENSG00000171631 | P2RY6 |  |  | 3257 | 4.058 | 0.3010 | Yes |
| 54 | ENSG00000101180 | HRH3 |  |  | 3338 | 3.953 | 0.3116 | Yes |
| 55 | ENSG00000259006 | AC092143.2 |  |  | 3463 | 3.690 | 0.3206 | Yes |
| 56 | ENSG00000158748 | HTR6 |  |  | 3616 | 3.482 | 0.3286 | Yes |
| 57 | ENSG00000126233 | SLURP1 |  |  | 3710 | 3.360 | 0.3372 | Yes |
| 58 | ENSG00000160716 | CHRNB2 |  |  | 3835 | 3.231 | 0.3448 | Yes |
| 59 | ENSG00000126895 | AVPR2 |  |  | 3848 | 3.210 | 0.3543 | Yes |
| 60 | ENSG00000126351 | THRA |  |  | 4014 | 3.007 | 0.3606 | Yes |
| 61 | ENSG00000163873 | GRIK3 |  |  | 4149 | 2.903 | 0.3671 | Yes |
| 62 | ENSG00000149403 | GRIK4 |  |  | 4199 | 2.861 | 0.3750 | Yes |
| 63 | ENSG00000150594 | ADRA2A |  |  | 4340 | 2.774 | 0.3810 | Yes |
| 64 | ENSG00000104826 | LHB |  |  | 4346 | 2.772 | 0.3893 | Yes |
| 65 | ENSG00000182687 | GALR2 |  |  | 4469 | 2.669 | 0.3953 | Yes |
| 66 | ENSG00000101405 | OXT |  |  | 4526 | 2.637 | 0.4023 | Yes |
| 67 | ENSG00000204982 | PRSS3P4 |  |  | 4543 | 2.637 | 0.4100 | Yes |
| 68 | ENSG00000131096 | PYY |  |  | 4595 | 2.617 | 0.4170 | Yes |
| 69 | ENSG00000121764 | HCRTR1 |  |  | 4651 | 2.584 | 0.4239 | Yes |
| 70 | ENSG00000164129 | NPY5R |  |  | 4706 | 2.551 | 0.4307 | Yes |
| 71 | ENSG00000244165 | P2RY11 |  |  | 4777 | 2.504 | 0.4371 | Yes |
| 72 | ENSG00000080293 | SCTR |  |  | 4849 | 2.469 | 0.4434 | Yes |
| 73 | ENSG00000175697 | GPR156 |  |  | 5054 | 2.375 | 0.4471 | Yes |
| 74 | ENSG00000149295 | DRD2 |  |  | 5122 | 2.338 | 0.4530 | Yes |
| 75 | ENSG00000164270 | HTR4 |  |  | 5165 | 2.314 | 0.4593 | Yes |
| 76 | ENSG00000157017 | GHRL |  |  | 5209 | 2.312 | 0.4656 | Yes |
| 77 | ENSG00000117971 | CHRNB4 |  |  | 5322 | 2.266 | 0.4705 | Yes |
| 78 | novel.231 | - |  |  | 5392 | 2.234 | 0.4761 | Yes |
| 79 | ENSG00000175591 | P2RY2 |  |  | 5427 | 2.223 | 0.4823 | Yes |
| 80 | ENSG00000124493 | GRM4 |  |  | 5434 | 2.220 | 0.4889 | Yes |
| 81 | ENSG00000100300 | TSPO |  |  | 5435 | 2.220 | 0.4956 | Yes |
| 82 | ENSG00000184523 | PTGER4P2 |  |  | 5490 | 2.203 | 0.5014 | Yes |
| 83 | ENSG00000180720 | CHRM4 |  |  | 5577 | 2.171 | 0.5065 | Yes |
| 84 | ENSG00000115353 | TACR1 |  |  | 5631 | 2.159 | 0.5121 | Yes |
| 85 | ENSG00000211451 | GNRHR2 |  |  | 5800 | 2.098 | 0.5156 | Yes |
| 86 | ENSG00000184160 | ADRA2C |  |  | 5832 | 2.085 | 0.5214 | Yes |
| 87 | ENSG00000064547 | LPAR2 |  |  | 5838 | 2.082 | 0.5276 | Yes |
| 88 | ENSG00000183971 | NPW |  |  | 5882 | 2.068 | 0.5331 | Yes |
| 89 | ENSG00000162009 | SSTR5 |  |  | 5926 | 2.055 | 0.5386 | Yes |
| 90 | ENSG00000248099 | INSL3 |  |  | 5943 | 2.050 | 0.5445 | Yes |
| 91 | ENSG00000004948 | CALCR |  |  | 6010 | 2.031 | 0.5495 | Yes |
| 92 | ENSG00000056291 | NPFFR2 |  |  | 6176 | 1.992 | 0.5528 | Yes |
| 93 | ENSG00000243831 | AL109933.2 |  |  | 6194 | 1.992 | 0.5585 | Yes |
| 94 | ENSG00000215644 | GCGR |  |  | 6292 | 1.977 | 0.5628 | Yes |
| 95 | ENSG00000113494 | PRLR |  |  | 6301 | 1.977 | 0.5687 | Yes |
| 96 | ENSG00000187848 | P2RX2 |  |  | 6519 | 1.920 | 0.5708 | Yes |
| 97 | ENSG00000101188 | NTSR1 |  |  | 6529 | 1.917 | 0.5764 | Yes |
| 98 | ENSG00000259169 | GNRHR2P1 |  |  | 6537 | 1.916 | 0.5821 | Yes |
| 99 | ENSG00000176884 | GRIN1 |  |  | 6857 | 1.851 | 0.5823 | Yes |
| 100 | ENSG00000183935 | HTR7P1 |  |  | 6918 | 1.835 | 0.5868 | Yes |
| 101 | ENSG00000274286 | ADRA2B |  |  | 6973 | 1.822 | 0.5914 | Yes |
| 102 | ENSG00000128285 | MCHR1 |  |  | 7004 | 1.816 | 0.5964 | Yes |
| 103 | ENSG00000169684 | CHRNA5 |  |  | 7121 | 1.788 | 0.5998 | Yes |
| 104 | ENSG00000213906 | LTB4R2 |  |  | 7161 | 1.782 | 0.6046 | Yes |
| 105 | ENSG00000161509 | GRIN2C |  |  | 7429 | 1.730 | 0.6052 | Yes |
| 106 | ENSG00000135744 | AGT |  |  | 7816 | 1.655 | 0.6037 | Yes |
| 107 | ENSG00000213903 | LTB4R |  |  | 7914 | 1.639 | 0.6070 | Yes |
| 108 | ENSG00000125787 | GNRH2 |  |  | 7951 | 1.631 | 0.6113 | Yes |
| 109 | ENSG00000197405 | C5AR1 |  |  | 7996 | 1.624 | 0.6155 | Yes |
| 110 | ENSG00000196811 | CHRNG |  |  | 8126 | 1.604 | 0.6181 | Yes |
| 111 | ENSG00000080644 | CHRNA3 |  |  | 8189 | 1.596 | 0.6219 | Yes |
| 112 | ENSG00000043591 | ADRB1 |  |  | 8208 | 1.593 | 0.6264 | Yes |
| 113 | ENSG00000105737 | GRIK5 |  |  | 8493 | 1.547 | 0.6263 | Yes |
| 114 | ENSG00000180616 | SSTR2 |  |  | 8618 | 1.532 | 0.6288 | Yes |
| 115 | ENSG00000257950 | P2RX5-TAX1BP3 |  |  | 8713 | 1.518 | 0.6318 | Yes |
| 116 | ENSG00000261762 | AC027228.2 |  |  | 8742 | 1.514 | 0.6359 | Yes |
| 117 | ENSG00000180914 | OXTR |  |  | 8844 | 1.501 | 0.6387 | Yes |
| 118 | ENSG00000184984 | CHRM5 |  |  | 8981 | 1.484 | 0.6409 | Yes |
| 119 | ENSG00000089041 | P2RX7 |  |  | 9047 | 1.474 | 0.6443 | Yes |
| 120 | ENSG00000106804 | C5 |  |  | 9106 | 1.466 | 0.6477 | Yes |
| 121 | ENSG00000148680 | HTR7 |  |  | 9511 | 1.415 | 0.6451 | Yes |
| 122 | ENSG00000179546 | HTR1D |  |  | 9619 | 1.402 | 0.6475 | Yes |
| 123 | ENSG00000171189 | GRIK1 |  |  | 9655 | 1.398 | 0.6512 | Yes |
| 124 | ENSG00000094755 | GABRP |  |  | 9743 | 1.390 | 0.6539 | Yes |
| 125 | ENSG00000125510 | OPRL1 |  |  | 10004 | 1.358 | 0.6536 | Yes |
| 126 | ENSG00000128310 | GALR3 |  |  | 10164 | 1.339 | 0.6549 | Yes |
| 127 | ENSG00000116014 | KISS1R |  |  | 10324 | 1.323 | 0.6562 | Yes |
| 128 | ENSG00000258839 | MC1R |  |  | 11101 | 1.256 | 0.6468 | No |
| 129 | ENSG00000168412 | MTNR1A |  |  | 11440 | 1.230 | 0.6448 | No |
| 130 | ENSG00000171388 | APLN |  |  | 11501 | 1.227 | 0.6475 | No |
| 131 | ENSG00000176136 | MC5R |  |  | 11549 | 1.223 | 0.6504 | No |
| 132 | ENSG00000164082 | GRM2 |  |  | 11653 | 1.214 | 0.6523 | No |
| 133 | ENSG00000116032 | GRIN3B |  |  | 11703 | 1.210 | 0.6551 | No |
| 134 | ENSG00000010438 | PRSS3 |  |  | 12292 | 1.157 | 0.6486 | No |
| 135 | ENSG00000169836 | TACR3 |  |  | 12912 | 1.107 | 0.6414 | No |
| 136 | ENSG00000197696 | NMB |  |  | 13778 | 1.043 | 0.6298 | No |
| 137 | ENSG00000163794 | UCN |  |  | 14031 | 1.023 | 0.6286 | No |
| 138 | ENSG00000267534 | S1PR2 |  |  | 14073 | 1.020 | 0.6310 | No |
| 139 | ENSG00000173080 | RXFP4 |  |  | 14111 | 1.017 | 0.6335 | No |
| 140 | ENSG00000151617 | EDNRA |  |  | 14285 | 1.004 | 0.6336 | No |
| 141 | ENSG00000170425 | ADORA2B |  |  | 14354 | 0.998 | 0.6354 | No |
| 142 | ENSG00000128271 | ADORA2A |  |  | 14382 | 0.996 | 0.6380 | No |
| 143 | ENSG00000283992 | SLURP2 |  |  | 14401 | 0.996 | 0.6407 | No |
| 144 | ENSG00000183454 | GRIN2A |  |  | 14553 | 0.996 | 0.6411 | No |
| 145 | ENSG00000164251 | F2RL1 |  |  | 14721 | 0.988 | 0.6413 | No |
| 146 | ENSG00000135124 | P2RX4 |  |  | 15083 | 0.965 | 0.6381 | No |
| 147 | ENSG00000213694 | S1PR3 |  |  | 15514 | 0.939 | 0.6336 | No |
| 148 | ENSG00000111886 | GABRR2 |  |  | 16106 | 0.913 | 0.6263 | No |
| 149 | ENSG00000184845 | DRD1 |  |  | 16190 | 0.907 | 0.6276 | No |
| 150 | ENSG00000170498 | KISS1 |  |  | 16280 | 0.901 | 0.6288 | No |
| 151 | ENSG00000108556 | CHRNE |  |  | 16594 | 0.882 | 0.6262 | No |
| 152 | ENSG00000050628 | PTGER3 |  |  | 17181 | 0.846 | 0.6187 | No |
| 153 | ENSG00000232354 | VIPR1-AS1 |  |  | 17286 | 0.839 | 0.6195 | No |
| 154 | ENSG00000114812 | VIPR1 |  |  | 17293 | 0.839 | 0.6219 | No |
| 155 | ENSG00000148498 | PARD3 |  |  | 17727 | 0.814 | 0.6170 | No |
| 156 | ENSG00000096395 | MLN |  |  | 18046 | 0.796 | 0.6140 | No |
| 157 | ENSG00000235481 | UBE2R2-AS1 |  |  | 19172 | 0.734 | 0.5971 | No |
| 158 | ENSG00000178623 | GPR35 |  |  | 19468 | 0.717 | 0.5942 | No |
| 159 | ENSG00000169860 | P2RY1 |  |  | 19518 | 0.714 | 0.5956 | No |
| 160 | ENSG00000163485 | ADORA1 |  |  | 19797 | 0.700 | 0.5930 | No |
| 161 | ENSG00000181104 | F2R |  |  | 19822 | 0.698 | 0.5947 | No |
| 162 | ENSG00000102539 | MLNR |  |  | 19898 | 0.694 | 0.5955 | No |
| 163 | ENSG00000113749 | HRH2 |  |  | 20158 | 0.680 | 0.5931 | No |
| 164 | ENSG00000169403 | PTAFR |  |  | 20159 | 0.679 | 0.5952 | No |
| 165 | ENSG00000112964 | GHR |  |  | 20349 | 0.668 | 0.5940 | No |
| 166 | ENSG00000145888 | GLRA1 |  |  | 20424 | 0.664 | 0.5947 | No |
| 167 | ENSG00000075073 | TACR2 |  |  | 20644 | 0.653 | 0.5930 | No |
| 168 | ENSG00000182771 | GRID1 |  |  | 20654 | 0.653 | 0.5948 | No |
| 169 | ENSG00000125910 | S1PR4 |  |  | 21389 | 0.615 | 0.5842 | No |
| 170 | ENSG00000175868 | CALCB |  |  | 21411 | 0.615 | 0.5857 | No |
| 171 | ENSG00000106018 | VIPR2 |  |  | 21583 | 0.603 | 0.5846 | No |
| 172 | ENSG00000169006 | NTSR2 |  |  | 21689 | 0.597 | 0.5846 | No |
| 173 | ENSG00000135914 | HTR2B |  |  | 21799 | 0.589 | 0.5845 | No |
| 174 | ENSG00000187474 | FPR3 |  |  | 22035 | 0.575 | 0.5823 | No |
| 175 | ENSG00000129749 | CHRNA10 |  |  | 22098 | 0.571 | 0.5830 | No |
| 176 | ENSG00000010310 | GIPR |  |  | 22169 | 0.566 | 0.5835 | No |
| 177 | ENSG00000116678 | LEPR |  |  | 22212 | 0.563 | 0.5845 | No |
| 178 | ENSG00000125730 | C3 |  |  | 22733 | 0.527 | 0.5772 | No |
| 179 | ENSG00000196689 | TRPV1 |  |  | 22858 | 0.517 | 0.5767 | No |
| 180 | ENSG00000145040 | UCN2 |  |  | 22894 | 0.514 | 0.5776 | No |
| 181 | ENSG00000169252 | ADRB2 |  |  | 22998 | 0.506 | 0.5774 | No |
| 182 | ENSG00000105464 | GRIN2D |  |  | 23523 | 0.476 | 0.5699 | No |
| 183 | ENSG00000171522 | PTGER4 |  |  | 24186 | 0.422 | 0.5599 | No |
| 184 | ENSG00000168830 | HTR1E |  |  | 24196 | 0.421 | 0.5610 | No |
| 185 | ENSG00000160951 | PTGER1 |  |  | 24202 | 0.420 | 0.5622 | No |
| 186 | ENSG00000170214 | ADRA1B |  |  | 24394 | 0.407 | 0.5602 | No |
| 187 | ENSG00000145451 | GLRA3 |  |  | 24424 | 0.405 | 0.5609 | No |
| 188 | ENSG00000244137 | AL512328.1 |  |  | 24434 | 0.404 | 0.5620 | No |
| 189 | ENSG00000198121 | LPAR1 |  |  | 24490 | 0.399 | 0.5623 | No |
| 190 | ENSG00000100739 | BDKRB1 |  |  | 24519 | 0.398 | 0.5630 | No |
| 191 | ENSG00000116329 | OPRD1 |  |  | 24551 | 0.395 | 0.5637 | No |
| 192 | ENSG00000113580 | NR3C1 |  |  | 24727 | 0.381 | 0.5618 | No |
| 193 | ENSG00000147437 | GNRH1 |  |  | 24962 | 0.358 | 0.5589 | No |
| 194 | ENSG00000127129 | EDN2 |  |  | 25407 | 0.316 | 0.5523 | No |
| 195 | ENSG00000166863 | TAC3 |  |  | 25429 | 0.313 | 0.5529 | No |
| 196 | ENSG00000151090 | THRB |  |  | 25448 | 0.311 | 0.5536 | No |
| 197 | ENSG00000152822 | GRM1 |  |  | 25513 | 0.307 | 0.5534 | No |
| 198 | ENSG00000071677 | PRLH |  |  | 25518 | 0.307 | 0.5543 | No |
| 199 | ENSG00000147246 | HTR2C |  |  | 25587 | 0.297 | 0.5540 | No |
| 200 | ENSG00000109738 | GLRB |  |  | 25801 | 0.271 | 0.5512 | No |
| 201 | ENSG00000168398 | BDKRB2 |  |  | 25823 | 0.268 | 0.5517 | No |
| 202 | ENSG00000065325 | GLP2R |  |  | 25878 | 0.261 | 0.5515 | No |
| 203 | ENSG00000144891 | AGTR1 |  |  | 26079 | 0.240 | 0.5488 | No |
| 204 | ENSG00000148926 | ADM |  |  | 26133 | 0.236 | 0.5487 | No |
| 205 | ENSG00000118432 | CNR1 |  |  | 26359 | 0.208 | 0.5455 | No |
| 206 | ENSG00000120251 | GRIA2 |  |  | 26530 | 0.188 | 0.5431 | No |
| 207 | ENSG00000136928 | GABBR2 |  |  | 26560 | 0.185 | 0.5432 | No |
| 208 | ENSG00000196639 | HRH1 |  |  | 26643 | 0.171 | 0.5423 | No |
| 209 | ENSG00000147145 | LPAR4 |  |  | 26727 | 0.160 | 0.5414 | No |
| 210 | ENSG00000164418 | GRIK2 |  |  | 26875 | 0.136 | 0.5393 | No |
| 211 | ENSG00000109163 | GNRHR |  |  | 26884 | 0.135 | 0.5396 | No |
| 212 | ENSG00000078401 | EDN1 |  |  | 26980 | 0.117 | 0.5383 | No |
| 213 | ENSG00000135346 | CGA |  |  | 27048 | 0.100 | 0.5375 | No |
| 214 | ENSG00000147571 | CRH |  |  | 27092 | 0.090 | 0.5370 | No |
| 215 | ENSG00000179097 | HTR1F |  |  | 27110 | 0.086 | 0.5370 | No |
| 216 | ENSG00000198785 | GRIN3A |  |  | 27116 | 0.085 | 0.5371 | No |
| 217 | ENSG00000125551 | PLGLB2 |  |  | 27157 | 0.070 | 0.5367 | No |
| 218 | ENSG00000110680 | CALCA |  |  | 27296 | 0.000 | 0.5343 | No |
| 219 | ENSG00000259384 | GH1 |  |  | 27411 | 0.000 | 0.5324 | No |
| 220 | ENSG00000145649 | GZMA |  |  | 27417 | 0.000 | 0.5323 | No |
| 221 | ENSG00000115138 | POMC |  |  | 27434 | 0.000 | 0.5320 | No |
| 222 | ENSG00000171860 | C3AR1 |  |  | 27550 | 0.000 | 0.5301 | No |
| 223 | ENSG00000187094 | CCK |  |  | 27612 | 0.000 | 0.5290 | No |
| 224 | ENSG00000226306 | NPY6R |  |  | 27737 | 0.000 | 0.5269 | No |
| 225 | ENSG00000118702 | GHRH |  |  | 27999 | 0.000 | 0.5225 | No |
| 226 | ENSG00000170820 | FSHR |  |  | 28080 | 0.000 | 0.5211 | No |
| 227 | ENSG00000101204 | CHRNA4 |  |  | 28094 | 0.000 | 0.5209 | No |
| 228 | ENSG00000175344 | CHRNA7 |  |  | 28257 | 0.000 | 0.5181 | No |
| 229 | ENSG00000279956 | AC073082.1 |  |  | 28290 | 0.000 | 0.5176 | No |
| 230 | ENSG00000167798 | C3P1 |  |  | 28442 | 0.000 | 0.5150 | No |
| 231 | ENSG00000132911 | NMUR2 |  |  | 28595 | 0.000 | 0.5124 | No |
| 232 | ENSG00000102239 | BRS3 |  |  | 28757 | 0.000 | 0.5097 | No |
| 233 | ENSG00000268089 | GABRQ |  |  | 28855 | 0.000 | 0.5080 | No |
| 234 | ENSG00000112164 | GLP1R |  |  | 28918 | 0.000 | 0.5070 | No |
| 235 | ENSG00000176358 | TAC4 |  |  | 28999 | 0.000 | 0.5056 | No |
| 236 | ENSG00000113262 | GRM6 |  |  | 29006 | 0.000 | 0.5055 | No |
| 237 | ENSG00000113889 | KNG1 |  |  | 29052 | 0.000 | 0.5048 | No |
| 238 | ENSG00000152034 | MCHR2 |  |  | 29244 | 0.000 | 0.5015 | No |
| 239 | ENSG00000144407 | PTH2R |  |  | 29393 | 0.000 | 0.4990 | No |
| 240 | ENSG00000106113 | CRHR2 |  |  | 29439 | 0.000 | 0.4982 | No |
| 241 | ENSG00000171051 | FPR1 |  |  | 29552 | 0.000 | 0.4963 | No |
| 242 | ENSG00000179603 | GRM8 |  |  | 29554 | 0.000 | 0.4963 | No |
| 243 | ENSG00000258691 | AL355102.2 |  |  | 29664 | 0.000 | 0.4944 | No |
| 244 | ENSG00000135312 | HTR1B |  |  | 29795 | 0.000 | 0.4922 | No |
| 245 | ENSG00000188822 | CNR2 |  |  | 29802 | 0.000 | 0.4921 | No |
| 246 | ENSG00000181195 | PENK |  |  | 29824 | 0.000 | 0.4918 | No |
| 247 | ENSG00000049247 | UTS2 |  |  | 29916 | 0.000 | 0.4902 | No |
| 248 | ENSG00000173198 | CYSLTR1 |  |  | 30124 | 0.000 | 0.4867 | No |
| 249 | ENSG00000138435 | CHRNA1 |  |  | 30127 | 0.000 | 0.4867 | No |
| 250 | novel.613 | - |  |  | 30166 | 0.000 | 0.4860 | No |
| 251 | ENSG00000136160 | EDNRB |  |  | 30205 | 0.000 | 0.4854 | No |
| 252 | ENSG00000232374 | GPR79 |  |  | 30588 | 0.000 | 0.4789 | No |
| 253 | ENSG00000171509 | RXFP1 |  |  | 30592 | 0.000 | 0.4788 | No |
| 254 | ENSG00000101327 | PDYN |  |  | 31007 | NaN | 0.4718 | No |
| 255 | ENSG00000165409 | TSHR |  |  | 31256 | NaN | 0.4676 | No |
| 256 | ENSG00000171049 | FPR2 |  |  | 31331 | NaN | 0.4664 | No |
| 257 | ENSG00000230386 | AC104057.1 |  |  | 31338 | NaN | 0.4663 | No |
| 258 | ENSG00000181072 | CHRM2 |  |  | 31450 | NaN | 0.4644 | No |
| 259 | ENSG00000157005 | SST |  |  | 31583 | NaN | 0.4622 | No |
| 260 | ENSG00000151834 | GABRA2 |  |  | 31866 | NaN | 0.4574 | No |
| 261 | ENSG00000006128 | TAC1 |  |  | 32283 | NaN | 0.4504 | No |
| 262 | ENSG00000132671 | SSTR4 |  |  | 32696 | NaN | 0.4434 | No |
| 263 | ENSG00000236136 | ADORA2BP1 |  |  | 32932 | NaN | 0.4394 | No |
| 264 | ENSG00000226820 | AL592049.1 |  |  | 32967 | NaN | 0.4389 | No |
| 265 | ENSG00000102287 | GABRE |  |  | 33202 | NaN | 0.4349 | No |
| 266 | ENSG00000166206 | GABRB3 |  |  | 33208 | NaN | 0.4349 | No |
| 267 | ENSG00000136305 | CIDEB |  |  | 33366 | NaN | 0.4322 | No |
| 268 | ENSG00000152208 | GRID2 |  |  | 33525 | NaN | 0.4296 | No |
| 269 | ENSG00000198822 | GRM3 |  |  | 33770 | NaN | 0.4255 | No |
| 270 | ENSG00000109158 | GABRA4 |  |  | 33826 | NaN | 0.4245 | No |
| 271 | ENSG00000102468 | HTR2A |  |  | 34195 | NaN | 0.4183 | No |
| 272 | ENSG00000146383 | TAAR6 |  |  | 34211 | NaN | 0.4181 | No |
| 273 | ENSG00000213218 | CSH2 |  |  | 34281 | NaN | 0.4169 | No |
| 274 | ENSG00000174417 | TRHR |  |  | 34507 | NaN | 0.4131 | No |
| 275 | ENSG00000168967 | PMCHL1 |  |  | 34632 | NaN | 0.4111 | No |
| 276 | ENSG00000237449 | TAAR4P |  |  | 34657 | NaN | 0.4107 | No |
| 277 | ENSG00000134640 | MTNR1B |  |  | 35927 | NaN | 0.3891 | No |
| 278 | ENSG00000265060 | PPY2P |  |  | 35931 | NaN | 0.3891 | No |
| 279 | ENSG00000022355 | GABRA1 |  |  | 36127 | NaN | 0.3858 | No |
| 280 | ENSG00000235998 | TAAR7P |  |  | 36178 | NaN | 0.3850 | No |
| 281 | ENSG00000266830 | AC061975.6 |  |  | 36213 | NaN | 0.3844 | No |
| 282 | ENSG00000141433 | ADCYAP1 |  |  | 36228 | NaN | 0.3842 | No |
| 283 | ENSG00000261561 | AC091304.5 |  |  | 36465 | NaN | 0.3802 | No |
| 284 | ENSG00000170989 | S1PR1 |  |  | 36833 | NaN | 0.3740 | No |
| 285 | ENSG00000120088 | CRHR1 |  |  | 37137 | NaN | 0.3689 | No |
| 286 | ENSG00000101200 | AVP |  |  | 37333 | NaN | 0.3656 | No |
| 287 | ENSG00000181977 | PYY3 |  |  | 37359 | NaN | 0.3652 | No |
| 288 | ENSG00000101958 | GLRA2 |  |  | 37590 | NaN | 0.3613 | No |
| 289 | ENSG00000204983 | PRSS1 |  |  | 37760 | NaN | 0.3585 | No |
| 290 | ENSG00000257852 | AC048344.2 |  |  | 37786 | NaN | 0.3581 | No |
| 291 | ENSG00000120903 | CHRNA2 |  |  | 37796 | NaN | 0.3579 | No |
| 292 | ENSG00000204174 | NPY4R |  |  | 37860 | NaN | 0.3569 | No |
| 293 | ENSG00000157219 | HTR5A |  |  | 37953 | NaN | 0.3554 | No |
| 294 | ENSG00000134443 | GRP |  |  | 37981 | NaN | 0.3549 | No |
| 295 | ENSG00000147432 | CHRNB3 |  |  | 38180 | NaN | 0.3516 | No |
| 296 | ENSG00000188778 | ADRB3 |  |  | 38268 | NaN | 0.3501 | No |
| 297 | ENSG00000186297 | GABRA5 |  |  | 38338 | NaN | 0.3490 | No |
| 298 | ENSG00000125522 | NPBWR2 |  |  | 38451 | NaN | 0.3471 | No |
| 299 | ENSG00000204681 | GABBR1 |  |  | 38781 | NaN | 0.3415 | No |
| 300 | ENSG00000136487 | GH2 |  |  | 39126 | NaN | 0.3357 | No |
| 301 | ENSG00000171517 | LPAR3 |  |  | 39202 | NaN | 0.3345 | No |
| 302 | ENSG00000185149 | NPY2R |  |  | 39209 | NaN | 0.3344 | No |
| 303 | ENSG00000122194 | PLG |  |  | 39515 | NaN | 0.3292 | No |
| 304 | ENSG00000122420 | PTGFR |  |  | 39781 | NaN | 0.3248 | No |
| 305 | ENSG00000172410 | INSL5 |  |  | 39884 | NaN | 0.3231 | No |
| 306 | ENSG00000108849 | PPY |  |  | 39973 | NaN | 0.3216 | No |
| 307 | ENSG00000146469 | VIP |  |  | 40133 | NaN | 0.3189 | No |
| 308 | ENSG00000159224 | GIP |  |  | 40188 | NaN | 0.3180 | No |
| 309 | ENSG00000166148 | AVPR1A |  |  | 40531 | NaN | 0.3122 | No |
| 310 | ENSG00000146385 | TAAR8 |  |  | 40629 | NaN | 0.3106 | No |
| 311 | ENSG00000277083 | PRSS3P3 |  |  | 40879 | NaN | 0.3064 | No |
| 312 | ENSG00000253403 | AC091944.1 |  |  | 41104 | NaN | 0.3026 | No |
| 313 | ENSG00000240935 | PLGLA |  |  | 41115 | NaN | 0.3025 | No |
| 314 | ENSG00000253179 | CALCP |  |  | 41143 | NaN | 0.3020 | No |
| 315 | ENSG00000170175 | CHRNB1 |  |  | 41211 | NaN | 0.3009 | No |
| 316 | ENSG00000124205 | EDN3 |  |  | 41230 | NaN | 0.3007 | No |
| 317 | ENSG00000155511 | GRIA1 |  |  | 41605 | NaN | 0.2943 | No |
| 318 | ENSG00000184303 | DRD5P1 |  |  | 41839 | NaN | 0.2904 | No |
| 319 | ENSG00000161610 | HCRT |  |  | 42202 | NaN | 0.2842 | No |
| 320 | ENSG00000169676 | DRD5 |  |  | 42492 | NaN | 0.2794 | No |
| 321 | ENSG00000225965 | AL117339.2 |  |  | 42553 | NaN | 0.2784 | No |
| 322 | ENSG00000139574 | NPFF |  |  | 42555 | NaN | 0.2784 | No |
| 323 | ENSG00000146378 | TAAR2 |  |  | 42814 | NaN | 0.2740 | No |
| 324 | ENSG00000264717 | NPY4R2 |  |  | 43167 | NaN | 0.2681 | No |
| 325 | ENSG00000181631 | P2RY13 |  |  | 43188 | NaN | 0.2677 | No |
| 326 | ENSG00000174343 | CHRNA9 |  |  | 43811 | NaN | 0.2572 | No |
| 327 | ENSG00000168229 | PTGDR |  |  | 43879 | NaN | 0.2561 | No |
| 328 | ENSG00000163288 | GABRB1 |  |  | 44391 | NaN | 0.2474 | No |
| 329 | ENSG00000163285 | GABRG1 |  |  | 44394 | NaN | 0.2474 | No |
| 330 | ENSG00000136488 | CSH1 |  |  | 44483 | NaN | 0.2459 | No |
| 331 | ENSG00000237110 | TAAR9 |  |  | 44515 | NaN | 0.2454 | No |
| 332 | ENSG00000272297 | AC018709.1 |  |  | 44531 | NaN | 0.2452 | No |
| 333 | ENSG00000265179 | AP000894.2 |  |  | 44559 | NaN | 0.2448 | No |
| 334 | ENSG00000267670 | AC022098.2 |  |  | 44679 | NaN | 0.2428 | No |
| 335 | ENSG00000164220 | F2RL2 |  |  | 45119 | NaN | 0.2353 | No |
| 336 | ENSG00000232168 | P2RY10BP |  |  | 46394 | NaN | 0.2137 | No |
| 337 | ENSG00000151577 | DRD3 |  |  | 47171 | NaN | 0.2005 | No |
| 338 | ENSG00000152207 | CYSLTR2 |  |  | 47283 | NaN | 0.1986 | No |
| 339 | ENSG00000220575 | HTR5A-AS1 |  |  | 47498 | NaN | 0.1950 | No |
| 340 | ENSG00000100448 | CTSG |  |  | 47538 | NaN | 0.1944 | No |
| 341 | ENSG00000082556 | OPRK1 |  |  | 47732 | NaN | 0.1911 | No |
| 342 | ENSG00000275811 | HTR1DP1 |  |  | 47843 | NaN | 0.1893 | No |
| 343 | novel.387 | - |  |  | 48208 | NaN | 0.1831 | No |
| 344 | ENSG00000108405 | P2RX1 |  |  | 48300 | NaN | 0.1816 | No |
| 345 | ENSG00000145864 | GABRB2 |  |  | 48497 | NaN | 0.1783 | No |
| 346 | ENSG00000145863 | GABRA6 |  |  | 48499 | NaN | 0.1783 | No |
| 347 | ENSG00000179073 | TAAR3P |  |  | 48537 | NaN | 0.1777 | No |
| 348 | ENSG00000121853 | GHSR |  |  | 48800 | NaN | 0.1733 | No |
| 349 | ENSG00000275896 | PRSS2 |  |  | 48860 | NaN | 0.1723 | No |
| 350 | ENSG00000182631 | RXFP3 |  |  | 49217 | NaN | 0.1663 | No |
| 351 | ENSG00000112038 | OPRM1 |  |  | 49366 | NaN | 0.1638 | No |
| 352 | ENSG00000172179 | PRL |  |  | 49493 | NaN | 0.1617 | No |
| 353 | ENSG00000250591 | PRSS3P1 |  |  | 49582 | NaN | 0.1602 | No |
| 354 | ENSG00000133636 | NTS |  |  | 49908 | NaN | 0.1547 | No |
| 355 | ENSG00000263715 | LINC02210-CRHR1 |  |  | 50038 | NaN | 0.1525 | No |
| 356 | ENSG00000254254 | AC012349.1 |  |  | 50067 | NaN | 0.1521 | No |
| 357 | ENSG00000175658 | DRD5P2 |  |  | 50166 | NaN | 0.1504 | No |
| 358 | ENSG00000131808 | FSHB |  |  | 50308 | NaN | 0.1481 | No |
| 359 | ENSG00000147434 | CHRNA6 |  |  | 50802 | NaN | 0.1397 | No |
| 360 | ENSG00000122585 | NPY |  |  | 50825 | NaN | 0.1394 | No |
| 361 | ENSG00000260444 | AC138749.2 |  |  | 50834 | NaN | 0.1393 | No |
| 362 | ENSG00000276393 | AC105429.2 |  |  | 51242 | NaN | 0.1324 | No |
| 363 | ENSG00000198049 | AVPR1B |  |  | 51279 | NaN | 0.1318 | No |
| 364 | ENSG00000146399 | TAAR1 |  |  | 52111 | NaN | 0.1177 | No |
| 365 | ENSG00000270002 | AC022028.2 |  |  | 52216 | NaN | 0.1159 | No |
| 366 | ENSG00000125675 | GRIA3 |  |  | 52329 | NaN | 0.1140 | No |
| 367 | ENSG00000110148 | CCKBR |  |  | 52470 | NaN | 0.1117 | No |
| 368 | ENSG00000078549 | ADCYAP1R1 |  |  | 52483 | NaN | 0.1115 | No |
| 369 | ENSG00000168959 | GRM5 |  |  | 52542 | NaN | 0.1106 | No |
| 370 | ENSG00000273079 | GRIN2B |  |  | 52664 | NaN | 0.1085 | No |
| 371 | ENSG00000236822 | NMBR-AS1 |  |  | 52778 | NaN | 0.1066 | No |
| 372 | ENSG00000178473 | UCN3 |  |  | 52908 | NaN | 0.1045 | No |
| 373 | ENSG00000113327 | GABRG2 |  |  | 53381 | NaN | 0.0965 | No |
| 374 | ENSG00000163394 | CCKAR |  |  | 53573 | NaN | 0.0932 | No |
| 375 | ENSG00000183185 | GABRR3 |  |  | 53592 | NaN | 0.0930 | No |
| 376 | ENSG00000182256 | GABRG3 |  |  | 53748 | NaN | 0.0904 | No |
| 377 | ENSG00000166603 | MC4R |  |  | 54176 | NaN | 0.0831 | No |
| 378 | ENSG00000241881 | AC245088.2 |  |  | 54214 | NaN | 0.0825 | No |
| 379 | ENSG00000183729 | NPBWR1 |  |  | 54265 | NaN | 0.0817 | No |
| 380 | ENSG00000171136 | RLN3 |  |  | 54470 | NaN | 0.0782 | No |
| 381 | ENSG00000233073 | AC005009.2 |  |  | 54513 | NaN | 0.0776 | No |
| 382 | ENSG00000152266 | PTH |  |  | 54545 | NaN | 0.0771 | No |
| 383 | ENSG00000109991 | P2RX3 |  |  | 54589 | NaN | 0.0764 | No |
| 384 | ENSG00000137252 | HCRTR2 |  |  | 55211 | NaN | 0.0658 | No |
| 385 | ENSG00000124089 | MC3R |  |  | 55359 | NaN | 0.0633 | No |
| 386 | ENSG00000279239 | AL391099.1 |  |  | 55433 | NaN | 0.0621 | No |
| 387 | ENSG00000070031 | SCT |  |  | 55613 | NaN | 0.0591 | No |
| 388 | ENSG00000249518 | AC011352.2 |  |  | 55824 | NaN | 0.0556 | No |
| 389 | ENSG00000119973 | PRLHR |  |  | 55992 | NaN | 0.0528 | No |
| 390 | ENSG00000183395 | PMCH |  |  | 56070 | NaN | 0.0515 | No |
| 391 | ENSG00000206145 | P2RX6P |  |  | 56117 | NaN | 0.0507 | No |
| 392 | ENSG00000255288 | AL358944.1 |  |  | 56171 | NaN | 0.0499 | No |
| 393 | ENSG00000134200 | TSHB |  |  | 56289 | NaN | 0.0479 | No |
| 394 | ENSG00000146276 | GABRR1 |  |  | 56359 | NaN | 0.0467 | No |
| 395 | ENSG00000274903 | AL109933.3 |  |  | 56523 | NaN | 0.0440 | No |
| 396 | ENSG00000120907 | ADRA1A |  |  | 56605 | NaN | 0.0427 | No |
| 397 | ENSG00000133105 | RXFP2 |  |  | 56610 | NaN | 0.0426 | No |
| 398 | ENSG00000264145 | AC239859.4 |  |  | 56640 | NaN | 0.0421 | No |
| 399 | ENSG00000135569 | TAAR5 |  |  | 56676 | NaN | 0.0416 | No |
| 400 | ENSG00000115263 | GCG |  |  | 56850 | NaN | 0.0387 | No |
| 401 | ENSG00000183979 | NPB |  |  | 57176 | NaN | 0.0332 | No |
| 402 | ENSG00000130368 | MAS1 |  |  | 58263 | NaN | 0.0147 | No |
| 403 | ENSG00000169040 | PMCHL2 |  |  | 58560 | NaN | 0.0097 | No |
| 404 | ENSG00000196277 | GRM7 |  |  | 58855 | NaN | 0.0047 | No |
| 405 | ENSG00000205035 | AC136759.1 |  |  | 59053 | NaN | 0.0014 | No |
Table: GSEA details [plain text format]

  

Fig 2: NEUROACTIVE\_LIGAND\_RECEPTOR\_INTERACTION(HSA04080)      
 Blue-Pink O' Gram in the Space of the Analyzed GeneSet

  

Fig 3: NEUROACTIVE\_LIGAND\_RECEPTOR\_INTERACTION(HSA04080): Random ES distribution      
 Gene set null distribution of ES for **NEUROACTIVE\_LIGAND\_RECEPTOR\_INTERACTION(HSA04080)**
